# Subjective Visual Vertical and Vestibular Evoked Myogenic Potential in Meniere's disease^☆^^☆☆^

**DOI:** 10.1016/j.bjorl.2022.11.005

**Published:** 2022-12-16

**Authors:** Maristela Mian Ferreira, Karen de Carvalho Lopes, Thaís Alvares de Abreu e Silva Grigol, Maurício Malavasi Ganança, Heloisa Helena Caovilla

**Affiliations:** Universidade Federal de São Paulo (UNIFESP), Escola Paulista de Medicina (EPM), Discipline of Otology and Neurotology, São Paulo, SP, Brazil

**Keywords:** Labyrinth diseases, Meniere's disease, Saccule and utricle, Vestibular function tests, Vestibular evoked myogenic potentials

## Abstract

**Objective:**

To evaluate otolith function by comparing the findings of the Subjective Visual Vertical, the cervical Vestibular Evoked Myogenic Potential and the ocular Vestibular Evoked Myogenic Potential tests in patients in the inter-crisis period of unilateral definite Meniere's disease.

**Methods:**

The sample consisted of an experimental group (n = 22; 10 men and 12 women, mean age 47.32 ± 12.82 years) with definite unilateral Meniere's disease and a control group (n = 14; 5 men and 9 women, with a mean age of 41.64 ± 13.45 years). They all underwent vestibular evaluation by means of Subjective Visual Vertical with the bucket method and, cervical and ocular Vestibular Evoked Myogenic Potential tests. The data were collected and subjected to statistical analysis.

**Results:**

The results of the comparison of the Subjective Visual Vertical, the cervical Vestibular Evoked Myogenic Potential, the ocular Vestibular Evoked Myogenic Potential and, the association of cervical Vestibular Evoked Myogenic Potential with the ocular Vestibular Evoked Myogenic Potential showed no significant difference, indicating concordance among the tests.

**Conclusion:**

The identified abnormalities and the concordance between the combined proportion of the Subjective Visual Vertical, the cervical Vestibular Evoked Myogenic Potential and the ocular Vestibular Evoked Myogenic Potential findings indicate that the association of these three tests contributes to the identification of sustained and transient otolith dysfunction in the inter-crisis of unilateral definite Meniere’s disease.

**Level of evidence:**

2.

## Highlights

• SVV test evaluates the static otolith system and, cervical and ocular VEMPs, examines the dynamic.
• SVV, with the bucket method, and cervical and ocular VEMPs, contribute to the assessment of vestibulopathies.
• SVV and cervical and ocular VEMPs together, are advantageous to assess otolith disorders in Meniere’s disease.

## Introduction

Procedures that explore the otolith macula function, such as the clinical evaluation of the perception of the Subjective Visual Vertical (SVV) and the Vestibular Evoked Myogenic Potential (VEMP)^1^, ^2^ have been included in the neurotological evaluation.

The VVS test examine the static or sustained otolith system, while the two types of VEMP, the cervical VEMP (cVEMP) and the ocular VEMP (oVEMP) tests, assess the dynamic or transient otolith system.^3^, ^4^, ^5^

When assessed by the SVV^6^, ^7^ with the bucket method, individuals indicate the vertical position of a straight line.^8^ With respect to VEMP test, sound stimuli generate short-latency biphasic potentials by activating otolith regions.^9^

In acute or chronic unilateral vestibulopathies, SVV and oVEMP alterations suggest the involvement of the utricular macula, whereas cVEMP abnormalities suggest the involvement of the saccular macula.^5^ Otolith loss interrupts the neural interaction, causing disorientation and postural instability.^3^, ^6^

SVV, cVEMP, and oVEMP abnormalities help in the functional assessment of Meniere’s Disease (MD),^2^ that is a disease characterized by episodic vertigo, hearing loss, tinnitus, and ear fullness, related to anatomical and vascular disorders, genetic predisposition, autoimmunity, or allergy.^10^, ^11^ Endolymphatic hydrops, believed to be a histopathological substrate of MD,^12^ is usually more frequent in the cochlea and saccule, involving the utricle and the three semicircular canals^13^; alone, it does not explain all the characteristics of the condition.^14^

The analysis of the concomitant results of SVV with the bucket method, cVEMP and oVEMP in the otolith functional evaluation in the inter-crisis of unilateral definite MD justified this research.

The aim of this study was to evaluate the otolith function comparing the results of SVV, cVEMP and oVEMP tests in the inter-crisis of unilateral definite MD.

## Methods

This analytical cross-sectional observational study was carried out at Discipline of Otology and Neurotology, Department of Otorhinolaryngology & Head and Neck Surgery, Universidade Federal de São Paulo – Escola Paulista de Medicina, between 2017 and 2021, with the approval of the Research Ethics Committee, under number 2.264.292/2017/CAAE: 70245617.1.0000.5505. The participants received information about the research and its objectives through an explanatory letter and signed the Free and Informed Consent form.

An experimental group (EG) and a control group (CG) were included.

The EG included patients of both genders, between 18 and 60 years of age, with a medical diagnosis of unilateral definite MD in the inter-crisis^10^: two or more vertigo episodes lasting from 20 min to 12 h, sensorineural hearing loss at low and medium frequencies and floating hearing loss, tinnitus and/or ear fullness; not better explained by another vestibular diagnosis; after the exclusion of other causes.^10^ This criterion does not use protocols for the functional assessment of the vestibular system; MD was diagnosed by clinical diagnosis based on symptoms reported by the patient and audiometric data; the differential diagnosis included transient ischemic attack, vestibular migraine, vestibular paroxysmia, recurrent unilateral vestibulopathy and other vestibular disorders. Magnetic resonance imaging can be used to exclude vestibular schwannoma or endolymphatic sac tumor when necessary; migraine, benign paroxysmal positional vertigo, and some systemic autoimmune conditions were considered comorbidities, as they cannot justify the symptoms of MD^10^; complaint/clinical history, physical examination alterations, and/or alterations in complementary tests (metabolic, autoimmunity, and/or or imaging) helped in the differential diagnosis with MD.

The affected ear was identified by hearing loss.

The CG comprised healthy volunteers from the community, homogeneous in terms of age and gender in relation to the EG, with no neurotological complaints or personal history.

Patients and volunteers with a history of head trauma, otological disorders, association with other vestibulopathies, neurological and psychiatric diseases, cervical limitations, inability to understand verbal commands, severe visual impairment or impairment not compensated by corrective lenses, conductive hearing loss at pure tone audiometry, and/or type B or C tympanometric curves, and alcohol intake 24 h before the evaluation were excluded.

The anamnesis, otoscopy, audiometry analysis (AD 227b Audiometer Interacoustics) and bioimpedance analysis (AT235 | Impedance audiometer, Interacoustics), SVV, cVEMP and oVEMP were performed on the same day.

The most recent audiometry characterized the hearing loss in stages, according to the average of the auditory thresholds at the frequencies of 500 Hz, 1 kHz, 2 kHz and 3 kHz: stage 1, values smaller than or equal to 25 dBHL; stage 2, between 26 and 40 dBHL; stage 3, from 41 to 70 dBHL and stage 4, greater than 70 dBHL.^15^

SVV was assessed by the bucket test.^16^, ^17^ A straight fluorescent strip on the inside bottom of an opaque bucket was aligned with the zero mark of a protractor on the outside bottom of the bucket and with the true vertical relative to the Earth.

The evaluation was performed in a semi-dark room. Participants sat with their heads upright inside the bucket to cover the peripheral vision and avoid visual cues. They were instructed to look at a fluorescent line and say “stop” when it reached the vertical position. The bucket was randomly rotated by the examiner, five times clockwise and five times counterclockwise.

Angular deviations from verticality were measured in degrees on the external bucket scale. True SVV was considered as zero degree. The difference between the final position and the true vertical configured the SVV value. The final value of the SVV was determined by the average of the absolute values in degrees in the ten attempts. Regarding the participant, clockwise deviations from zero (to the right) were considered positive and counterclockwise ones (to the left), negative. The final slope direction was determined by the sum of the values of the ten attempts, considering the positive or negative sign^18^; the sum of the deviations to the right and left equal to zero defined the absence of directional prevalence.

cVEMP and oVEMP were performed with ICS Chartr EP 200 equipment (GN-Otometrics). The capture of responses in cVEMP and oVEMP and the control of electromyographic activity in cVEMP used surface electrodes, after dermabrasion of the standardized test sites; maximum electrode impedance was 5k ohms.

In cVEMP, the ground electrode was placed in the frontal midline; the active electrodes, on the upper half of the right and left sternocleidomastoid muscles; the electrodes for the electromyographic control were placed below the active ones; and the reference electrode on the manubrium of the sternum. The patients remained in the sitting position, with cervical rotation contralateral to the stimulated ear; the captured and analyzed responses were ipsilateral to the sound stimulus (stimulated ear). The acquired responses consisted of a biphasic potential with the first positive wave, p13 (P1) and the second negative wave, n23 (N1).

For the oVEMP test, the ground electrode was placed on the frontal midline; the active electrodes were positioned on the inferior orbital margin in the pupillary line; and the reference electrodes were placed below the active ones. The patients, while sitting on a chair, were instructed to look at a fixed point positioned 30 ° above the eye line. The captured and analyzed responses were contralateral to the sound stimulus (stimulated ear) and consisted of a biphasic potential with the first negative wave, n10 (N1) and the second positive wave, p15 (P1).

Insert earphones were used for the cVEMP test in order to present the tone burst sound stimulus (duration: rise: 1.50 ms; plateau: 0.0 ms; fall: 1.50 ms) of 500 Hz, with an average of 150 stimuli, rarefied polarity and intensity of 95 dBHL. The analysis window was 53.3 ms with 10 Hz high-pass and 1500 Hz low-pass filters.^19^

For the oVEMP, the tone burst sound stimulus (duration: rise: 1.50 ms; plateau: 0.0 ms; fall: 1.50 ms) of 500 Hz, with alternating polarity, with an average of 150 stimuli and intensity of 95 dBHL was presented through insert earphones. The analysis window was 53.3 ms with a high-pass filter of 10 Hz and a low-pass filter of 1500 Hz.^20^

Three reproducible tracings were performed for cVEMP and oVEMP; the two best traces were chosen and the weight sum was performed, obtaining a third record used for the analysis.^21^, ^22^ In the cVEMP record, electromyographic responses between 50 and 200 μv were accepted, to maintain the adequate and constant level of contraction.^23^, ^24^

The analyzed cVEMP and oVEMP parameters were presence or absence of the biphasic potential, absolute p13latency values (P1), n23 (N1), n10 (N1) and p15 (P1), p13-n23 (P1‒N1) and n10-p15 (N1‒P1) inter-amplitude and asymmetry index.

The data were tabulated and submitted to descriptive statistical analysis using mean, median, minimum and maximum values, standard deviation, absolute and relative frequencies. The unresponsive ears were excluded for the analysis of the P1 and N1 absolute latencies and the inter-amplitude of the VEMPs. The asymmetry index was calculated when there was a response in at least one ear.

The SVV analyses were performed by the EG and CG and the cVEMP and oVEMP analyses considered Symptomatic (SE), Asymptomatic (AE) and Control (CE) ears. The normal or abnormal results of the SVV, cVEMP and oVEMP parameters were interpreted by comparison with reference values determined by the CG, calculated as the mean ± 2 Standard Deviations (SD). Increased mean SVV values, absence of biphasic potential, increased P1 and N1 latency, decreased inter-amplitude and asymmetry index above the upper limit were considered as altered responses.

The Shapiro–Wilk, Levene, Mann–Whitney, Fisher, ANOVA, Student's *t*, Kruskal–Wallis, and McNemar statistical tests were used, depending on the characteristics of the sample data. The effect size of differences between groups was assessed by calculating Cohen's *d* or Rosenthal’s *r* coefficients. The calculation of 95% Confidence Intervals used the bias-correction and accelerated method based on 2000 bootstrap samples. The statistical significance value adopted was less than or equal to 5% (*p* ≤ 0.05). The SPSS Statistics software, version 25.0 (IBM Corp, Armonk, NY, USA) was used in the statistical analysis.

## Results

Of 330 patients with clinical suspicion of MD, evaluated at the MD outpatient clinic, 270 cases were not included in the research because they did not meet the inclusion criteria; 32 refused to participate in the study; 28 accepted, but six did not show up on the scheduled day.

The sample consisted of 22 patients with MD (EG), 10 (45.45%) males and 12 (54.55%) females, aged between 19 and 60 years, with a mean age of 47.32 ± 12, 82 years (x ± SD) and 14 healthy individuals (CG), 5 (35.71%) males and 9 (64.29%) females, aged between 18 and 60 years, with a mean age of 41.64 ± 13.45 years. There was no statistically significant difference between the EG and CG regarding gender (*p* = 0.732) and age (*p* = 0.167). The right ear was involved in nine (40.91%) cases and the left in 13 (59.09%). As for hearing loss, five (22.73%) cases were classified as stage 1; six (27.27%) as 2; nine (40.91%) as 3 and two (9.09%) as stage 4. The duration of MD ranged from four months to 20 years (mean of 5.94 years).

Table 1 shows the significant difference between the EG and the CG regarding the mean SVV: the EG had a higher value than the CG. The reference value calculated for the SVV, considering the 14 individuals in the CG, was 2.37°.

**Table 1:** Descriptive values and comparative analysis of the experimental groups of patients with Meniere's disease (n = 22) and controls (n = 14) regarding the mean absolute values in degrees of the Subjective Visual Vertical (SVV).

SVV	Group	n	Mean	SD	Median	Min.	Max.	*p*	ES
Mean (°)	CG	14	1.29 (1.03–1.55)	0.54	1.45 (1.05–1.50)	0.40	2.40	**<0.001**^a^**,**^b^	2.766*^d^*
	EG	22	2.77 (2.23–3.34)	1.26	2.55 (1.90–3.40)	0.70	5.50		

Student's *t* test for independent samples with post-hoc analysis using the Games-Howell test; calculation of the effect size using the *d* coefficient.

*Note*: Values in square brackets indicate the upper and lower limits of the 95% confidence intervals.

SVV, Subjective Visual Vertical; SD, standard deviation; Min., minimum; Max., maximum; CG, control group; EG, experimental group; ES, effect size.

a Statistically significant value at the 5% level (*p* ≤ 0.05).

b Calculated with Welch correction for heteroscedasticity.

Table 2 shows the significant difference between the EG and the CG in relation to normal and altered SVV results: the EG had a higher proportion of altered results than the CG. Of the 13 (59.1%) patients in the EG with abnormal deviation of the SVV, two (15.4%) occurred in the direction of the SE and 11 (84.6%) in the direction of the AE.

**Table 2:** Comparison between the experimental group of patients with Meniere’s disease (n = 22) and controls (n = 14) in relation to the result of the Subjective Visual Vertical (SVV).

SVV	Result	Group						*p*-Value
		CG		EG		Total		
		n	%	n	%	n	%	
Mean	Altered	1	7.14	13	59.09	14	38.89	**<0.001**^a^
	Normal	13	92.86	9	40.91	22	61.11	

Fisher’s exact test.

CG, control group; EG, experimental group.

a Statistically significant value at the 5% level (*p* ≤ 0.05).

The CG showed responses to cVEMP and oVEMP in 28 ears. In the EG, the absence of response occurred in four SE and in three AE in cVEMP and in nine SE and six AE in oVEMP.

Table 3 shows the significant difference between the groups of ears in relation to inter-amplitude; the post-hoc analysis showed a significant difference between SE and CE (*p* = 0.008, r = 0.442), with a lower value for SE in cVEMP.

**Table 3:** Descriptive values and comparative analysis of symptomatic (n = 18) and asymptomatic (n = 19) ears in the experimental group of patients with Meniere’s disease and ears of individuals in the control group (n = 28) in relation to the P1 and N1 absolute latencies and inter-amplitude, and between the experimental (n = 21) and control (n = 14) groups, regarding the cVEMP asymmetry index.

cVEMP	Group	n	Mean	SD	Median	Min.	Max.	*p*	ES
P1 Latency (ms)	SE	18	15.24 (13.90–16.70)	3.07	14.21 (12.67–17.83)	11.33	21.50	0.971^b^	‒
	AE	19	14.70 (13.69–15.84)	2.47	14.17 (12.92–15.33)	12.08	19.83		
	CE	28	14.54 (13.91–15.17)	1.71	14.90 (14.45–15.17)	11.17	17.67		
N1 Latency (ms)	SE	18	23.43 (21.95–25.10)	3.36	22.75 (21.08–24.75)	19.25	30.67	0.256^a^	‒
	AE	19	23.50 (22.31–24.74)	2.78	23.08 (21.58–25.33)	19.00	27.67		
	CE	28	22.29 (21.36–23.17)	2.44	22.75 (21.66–23.50)	17.67	26.50		
Inter-amplitude (μV)	SE	18	88.75 (70.39. ‒109.44)	48.49	72.04 (58.85–104.06)	26.63	191.50	0.011^b,^^d^	‒
	AE	19	136.01 (100.60–179.47)	98.48	94.67 (80.25‒44.24)	46.34	445.86		
	CE	28	124.26 (111.57–137.35)	33.36	123.0 (104.54‒37.00)	69.08	190.00		
Asymmetry index	CG	14	22.28 (18.96–25.94)	6.41	21.53 (17.72–27.49)	14.11	36.12	0.538^c^	0.108*^r^*
	EG	21	41.92 (27.39–57.23)	36.81	27.29 (14.29–53.76)	1.95	100		

ANOVA with independent factor (^a^), Kruskal–Wallis test (^b^) with post-hoc analysis of Dunn's test with Bonferroni correction; calculation of the effect size using the r coefficient; Mann–Whitney *U* test (^c^).

*Note*: Values in square brackets indicate the upper and lower limits of the 95% confidence intervals.

cVEMP, cervical Vestibular Evoked Myogenic Potential; SE, symptomatic ears; AE, asymptomatic ears; CE, control ears; EG, experimental group; CG, control group; SD, standard deviation; Min., minimum; Max., maximum; ES, effect size.

d Statistically significant value at the 5% level (*p* ≤ 0.05).

Table 4 shows the significant difference between the EG and CG regarding the asymmetry index: the EG had a higher value compared to the CG in the oVEMP.

**Table 4:** Descriptive values and comparative analysis of symptomatic (n = 13) and asymptomatic (n = 16) ears in the experimental group of patients with Meniere’s disease and ears of individuals in the control group (n = 28) in relation to the N1 and P1 absolute latencies and inter-amplitude, and between the experimental (n = 16) and control (n = 14) groups, regarding the asymmetry index in the oVEMP.

oVEMP	Group	n	Mean	SD	Median	Min.	Max.	*p*	E.S.
N1 Latency (ms)	SE	13	10.26 (9.81–10.58)	0.71	10.42 (10.17–10.50)	8.16	11.08	0.195^a^	‒
	AE	16	10.50 (10.30–10.69)	0.47	10.50 (10.46–10.50)	9.42	11.58		
	CE	28	10.10 (9.87‒10.33)	0.78	10.11 (9.75–10.17)	8.90	11.42		
P1 Latency (ms)	SE	13	14.48 (13.87–15.03)	1.14	14.75 (14.75‒14.75)	12.08	15.92	0.600^a^	‒
	AE	16	14.24 (14.34–15.00)	2.59	14.75 (14.34–15.00)	5.00	16.67		
	CE	28	14.34 (13.95–14.71)	1.14	13.97 (13.74–15.42)	12.07	16.09		
Inter-amplitude (μV)	SE	13	6.26 (3.80–9.03)	4.38	4.42 (2.49–8.46)	1.36	15.16	0.972^a^	‒
	AE	16	6.06 (4.64–7.52)	3.10	5.94 (3.82–7.46)	2.10	11.15		
	CE	28	5.83 (5.15–6.54)	2.35	5.44 (4.22–6.05)	3.07	12.00		
Asymmetry index	CG	14	16.27 (12.63–20.14)	8.26	13.94 (9.74‒20.51)	5.24	33.33	**0.015**^b,^^c^	0.440^r^
	EG	16	41.26 (26.76–57.64)	32.86	28.80 (18.24–58.48)	6.63	100		

Kruskal–Wallis test (^a^); Mann–Whitney *U* test (^b^); coefficient r (^r^).

*Note*: Values in square brackets indicate the upper and lower limits of the 95% confidence intervals.

oVEMP, ocular Vestibular Evoked Myogenic Potential; SE, symptomatic ears; AE, asymptomatic ears; CE, control ears; EG, experimental group; CG, control group; SD, standard deviation; Min., minimum; Max., maximum; ES, effect size.

c Statistically significant value at the 5% level (*p* ≤ 0.05).

Considering the CG individuals, the reference values for P1, N1 absolute latencies, inter-amplitude and asymmetry index were 17.96 ms; 27.17 ms; 57.54 μV and 35.10% for cVEMP; and, 11.66 ms; 16.62 ms; 1.13 μV and 32.79% for oVEMP

Table 5 shows the significant difference between altered or normal results of SE, AE and CE regarding P1 latency, N1 latency and cVEMP inter-amplitude in the post-hoc analysis. The SE showed a higher proportion of altered P1 latency and inter-amplitude results compared to CE and a significant difference between altered or normal results regarding the asymmetry index of the EG and the CG, with the EG showing a higher proportion of asymmetric results.

**Table 5:** Comparison of altered or normal results of symptomatic (n = 18 for cVEMP and n = 13 for oVEMP) and asymptomatic (n = 19 for cVEMP and n = 16 for oVEMP) ears in the group of patients with Meniere’s disease and ears from individuals from the control group (n = 28) in relation to the absolute N1 and P1 latencies and inter-amplitude, and between the experimental (n = 21 for cVEMP and n = 16 for oVEMP) and control (n = 14) groups, regarding the asymmetry index in the cVEMP and oVEMP.

Test	Parameter	Group	Altered		Normal		Total		*p*
			n	%	n	%	n	%	
cVEMP	Latency P1 (ms)	SE	4	22.22^a^	14	77.78^a^	18	100	**0.014^c,^**^e^
		AE	3	15.79^a,b^	16	84.21^a,b^	19	100	
		CE	0	0.00^b^	28	100^b^	28	100	
	Latency N1 (ms)	SE	3	16.67^a^	15	83.33^a^	18	100	**0.040^c,^**^e^
		AE	3	15.79^a^	16	84.21^a^	19	100	
		CE	0	0.00^a^	28	100^a^	28	100	
	Inter-amplitude (μV)	SE	5	27.78^a^	13	72.22^a^	18	100	**0.007^c,^**^e^
		AE	2	10.53^a,b^	17	89.47^a,b^	19	100	
		CE	0	0.00^b^	28	100^b^	28	100	
	Asymmetry Index (%)	CG	1	7.14	13	92.86	14	100	**0.023^d,^**^e^
		EG	10	47.62	11	52.38	21	100	
oVEMP	N1 Latency (ms)	SE	0	0.00	13	100	13	100	NC
		AE	0	0.00	16	100	16	100	
		CE	0	0.00	28	100	28	100	
	P1 Latency (ms)	SE	0	0.00	13	100	13	100	0.509
		AE	1	6.25	15	93.75	16	100	
		CE	0	0.00	28	100	28	100	
	Inter-amplitude (μV)	SE	0	0.00	13	100	13	100	NC
		AE	0	0.00	16	100	16	100	
		CE	0	0.00	28	100	28	100	
	Asymmetry Index (%)	CG	1	7.14	13	92.86	14	100	0.086
		EG	6	37.50	10	62.50	16	100	

Fisher’s exact test with post-hoc analysis using the z (^c^) test; Fisher's exact test (^d^).

*Note*: Each superscript letter (^a,b^) indicates a proportion of a column that showed a statistically significant difference from the others in the same line and with different letters.

cVEMP, cervical Vestibular Evoked Myogenic Potential; oVEMP, ocular Vestibular Evoked Myogenic Potential; SE, symptomatic ears; AE, asymptomatic ears; CE, control ears; CG, control group; EG, experimental group; NC, not calculable.

e Statistically significant value at the 5% level (*p* ≤ 0.05).

Of the 22 patients with MD, eight had cVEMP within normal parameters; SE abnormalities occurred in 11 cases, including absent responses in four, increased P1 absolute latency in four and N1 latency in three, and decreased inter-amplitude in five. AE abnormalities occurred in nine cases, including absent responses in three, increased P1 absolute latency in three and N1 latency in two, and decreased inter-amplitude in two; the increase in the asymmetry index occurred in ten cases.

Of the 22 patients with MD, 12 had oVEMP within normal parameters, nine showed absent responses in the SE; seven showed abnormalities in the AE, including absent responses in six, increased P1 absolute latency in one; and increased asymmetry index in six.

Table 6 shows a non-significant difference between the results of SVV and cVEMP, oVEMP and cVEMP + oVEMP: there was no disagreement between the SVV results and the isolated or associated VEMP results.

**Table 6:** Comparison of the overall result of cVEMP, oVEMP and cVEMP + oVEMP with the altered or normal SVV finding per individual, in the 22 patients with Meniere’s disease.

Test	Category	SVV				*p*
		Altered		Normal		
		n	%	n	%	
cVEMP	Altered	11	50.00	6	27.27	0.289
	Normal	2	9.09	3	13.64	
oVEMP	Altered	7	31.82	5	22.73	>0.999
	Normal	6	27.27	4	18.18	
cVEMP + oVEMP	Altered	7	46.66	4	26.66	0.414
	Normal	2	13.33	2	13.33	

McNemar test.

cVEMP, cervical Vestibular Evoked Myogenic Potential; oVEMP, ocular Vestibular Evoked Myogenic Potential; SVV, Subjective Visual Vertical.

Of the 22 patients in the EG, two (9.1%) showed normal SVV, cVEMP and oVEMP results; seven (31.8%) had alterations in the three tests, four (18.2%) in the SVV and cVEMP; four (18.2%) in the cVEMP and oVEMP; two (9.1%) in the SVV; two (9.1%) in the cVEMP, and one (4.5%) in the oVEMP.

## Discussion

The SVV, cVEMP and oVEMP tests evaluated the otolith function in 22 patients with definite unilateral MD in the inter-crisis period, with no gender predominance and age range varying from 19 and 60 years, similar to the controls, so that older age does not influence the acquisition of VEMPs.^25^ Prolonged N1 and P1 latencies and reduced oVEMP inter-amplitude occurred in patients aged 60 years and older^26^ and oVEMP N1 latency and cVEMP p13 and n23 latencies were also longer in the elderly than in young adults.^27^

MD is a multifactorial clinical condition intensely studied in the scientific literature; the underlying etiology remains unclear, but it is associated with increased endolymphatic volume in the inner ear, culminating in acute auditory and vestibular symptoms. Endolymphatic hydrops can affect the otolith structures; functional disorders of the saccule and utricle can be recognized by cVEMP, oVEMP^28^ and SVV.^29^

Hearing loss occurred in decreasing order of prevalence in stages 3; 2; 1 and 4, characterizing a predominance of moderate to severe auditory disorders, similar to findings reported in MD.^24^, ^30^, ^31^, ^32^, ^33^, ^34^, ^35^, ^36^

MD duration was highly variable, ranging from a few months to two decades, with an average of less than six years, comparable to the literature, although longer duration has also been reported.^24^, ^31^, ^32^, ^35^^,^^36^

The SVV assessed through the bucket method was identified in only five studies in MD.^37^, ^38^, ^39^, ^40^, ^41^

In this study, patients with MD showed a high proportion of SVV alterations; the abnormal deviation of the SVV predominated in the direction of the AE, but also occurred in the direction of the SE and for both ears. SVV alterations have also been mentioned in MD.^29^, ^37^, ^38^, ^42^^,^^43^ SVV deviation to the side of the lesion and to the opposite side in unilateral MD and without lateralization in bilateral MD has also been found.^30^ Abnormal inter-crisis SVV in vestibulopathies suggests insufficient vestibular compensation.^25^ Deviation to the symptomatic or asymptomatic side has also been observed in the acute period of MD.^29^, ^39^

An altered cVEMP occurred in most cases in this study. The absence of the biphasic potential was similar in the SE and AE in MD; the SE showed a decrease in inter-amplitude, a greater proportion of alterations in P1 latency, inter-amplitude and asymmetry index. Absent cVEMP is attributable to otolith hydrops,^30^ loss of saccular function, or impairment of the brainstem and cervical spinal cord pathways involved in response generation.^25^ Abnormalities in cVEMP in AE suggest that the AE could be compromised in the future.^41^ Alterations in cVEMP parameters, which vary from study to study, have also been reported in MD cases. Absent cVEMP was mentioned in SE and AE,^24^, ^30^, ^31^, ^32^, ^33^, ^34^, ^35^, ^36^, ^44^, ^45^, ^46^ prolonged P1 and/or N1 latencies,^24^, ^31^, ^32^, ^34^^,^^36^, ^44^, ^46^ decreased inter-amplitude,^24^, ^44^ and altered asymmetry index.^33^, ^35^, ^47^

In turn, altered oVEMP occurred in just over half of the number of cases in this study. There was a higher rate of asymmetry in the EG than in the CG; SE and AE showed a higher proportion of absent biphasic potential. The absence of oVEMP has been suggested as possibly due to otolith hydrops.^32^ Altered oVEMP parameters have also been mentioned in MD, with variable results in the literature. Absent VEMP was mentioned in SE and AE,^30^, ^33^, ^34^, ^35^, ^36^ as well as alterations in P1 and/or N1 latencies^32^, ^34^, ^36^, ^46^ and asymmetry index.^33^, ^35^, ^47^

According to the authors clinical experience, the criteria of biphasic potential absence and the magnitude of the potential are the most important, mainly for the functional evaluation of the involved peripheral structures (otolith organs and/or vestibular nerves). The magnitude of the potential is reflected in the size of the response, that is, its amplitude. Due to the intra and interindividual amplitude variability, the asymmetry index is the most frequently used in practice to analyze the magnitude of the potential, comparing the amplitude of the response between both ears of the same individual.

The associated assessment of SVV + oVEMP^48^, ^49^ and SVV + cVEMP has been described,^38^, ^40^, ^43^ but no publications were found that applied all three procedures in MD, which indicates an original aspect of this research.

In the 22 patients with MD, SVV + cVEMP + oVEMP, SVV + cVEMP, cVEMP + oVEMP, SVV, cVEMP and oVEMP abnormalities appeared in decreasing order of prevalence. More frequent alterations in cVEMP than in oVEMP suggest that saccular dysfunction is more common than the utricular dysfunction; ears with abnormal cVEMP and oVEMP may have severe hydrops, causing concomitant utricle and saccule dysfunction.^30^ In extensive damage to the utricular macula, responses from the sustained and transient systems cause alterations in SVV and oVEMP; when utricular injury only affects the transient function, only oVEMP will be altered; or if it affects only the sustained function, it affects SVV.^3^

In this study, the normal and altered results of SVV, cVEMP, oVEMP and cVEMP + oVEMP were concordant in patients with MD, showing that when SVV showed a higher proportion of abnormal results, isolated or associated VEMPs also showed a higher proportion of abnormalities and the same happened with the normal results. The concordance between the proportion of SVV and VEMP findings indicates that these three tests are appropriate to demonstrate saccular and utricular functional abnormalities in MD. Other studies have highlighted the lack of correlation between cVEMP and SVV, with more frequent alterations in saccular function in cVEMP^43^; between SVV and cVEMP, with twice as many changes in SVV^38^; and between the lateralization of the SVV deviation and the abnormal asymmetry index to oVEMP, showing that the association of these utricular function tests is not redundant and may show different otolith dysfunctions in MD.^49^

Although vestibular function tests are not recommended for the diagnosis of MD,^10^ they can provide information in the evaluation and follow-up of patients with atypical symptoms or difficulty in determining the affected ear, which may even be useful when considering surgery for this vestibulopathy.^9^, ^28^

In this research, the findings of the SVV and those of cVEMP and oVEMP show that the association of these three tests, which assess different regions of the saccular and utricular maculae, is advantageous for measuring the degree and extent of sensory impairment in MD.

Other studies associating SVV, cVEMP and oVEMP are necessary for a more comprehensive characterization of saccular and utricular dysfunctions and to corroborate the results of this experiment in MD and also in other vestibulopathies.

## Conclusion

The identified abnormalities and the concordance of the combined findings of SVV, cVEMP and oVEMP tests indicate that the association of these three tests contributes to the identification of sustained and transient otolith dysfunction in the inter-crisis of unilateral definite Meniere’s disease.

## Funding

Coordination Foundation for the Improvement of Higher Education Personnel – CAPES (Doctoral Scholarship).

## Conflicts of interest

The authors declare no conflicts of interest.
